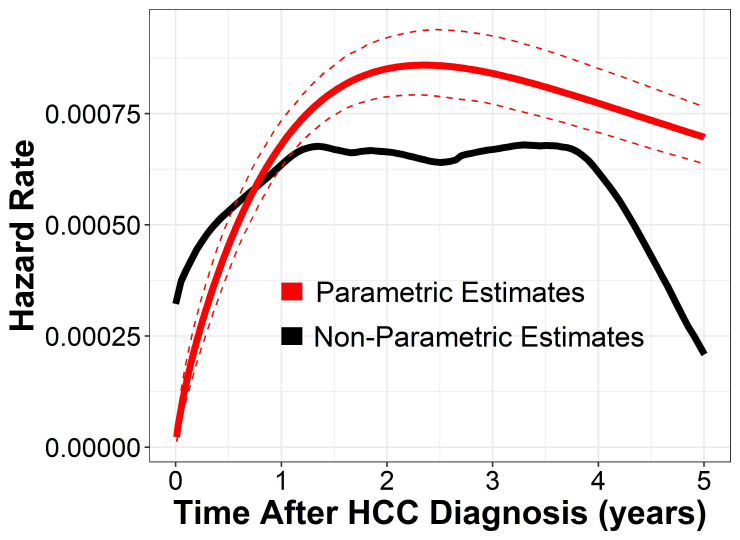# Predicting long-term survival among patients with HCC

**DOI:** 10.1097/HC9.0000000000000581

**Published:** 2024-11-04

**Authors:** David Goldberg, Peter P. Reese, David A. Kaplan, Yalda Zarnegarnia, Neelima Gaddipati, Sirisha Gaddipati, Binu John, Catherine Blandon

**Affiliations:** 1 Department of Medicine, Division of Digestive Health and Liver Diseases, University of Miami Miller School of Medicine, Miami, Florida, USA; 2 Department of Public Health Sciences, University of Miami Miller School of Medicine, Miami, Florida, USA; 3 Sylvester Comprehensive Cancer Center, University of Miami Miller School of Medicine, Miami, Florida, USA; 4 Department of Medicine, Renal-Electrolyte and Hypertension Division, University of Pennsylvania Perelman School of Medicine, Philadelphia, Pennsylvania, USA; 5 Department of Biostatistics, Epidemiology, and Informatics, University of Pennsylvania Perelman School of Medicine, Philadelphia, Pennsylvania, USA; 6 Division of Gastroenterology and Hepatology, Department of Medicine, University of Pennsylvania Perelman School of Medicine, Philadelphia, Pennsylvania, USA; 7 Department of Medicine, Corporal Michael J. Crescenz VA Medical Center, Philadelphia, Pennsylvania, USA; 8 Department of Medicine, Jackson Memorial Hospital, Miami, Florida, USA; 9 Department of Medicine, Bruce Carter VA Medical Center, Miami, Florida, USA

**Keywords:** HCC, prediction, survival

## Abstract

**Background:**

Prognosticating survival among patients with HCC and cirrhosis must account for both the tumor burden/stage, as well as the severity of the underlying liver disease. Although there are many staging systems used to guide therapy, they have not been widely adopted to predict patient-level survival after the diagnosis of HCC. We sought to develop a score to predict long-term survival among patients with early- to intermediate-stage HCC using purely objective criteria.

**Methods:**

Retrospective cohort study among patients with HCC confined to the liver, without major medical comorbidities within the Veterans Health Administration from 2014 to 2023. Tumor data were manually abstracted and combined with clinical and laboratory data to predict 5-year survival from HCC diagnosis using accelerated failure time models. The data were randomly split using a 75:25 ratio for training and validation. Model discrimination and calibration were assessed and compared to other HCC staging systems.

**Results:**

The cohort included 1325 patients with confirmed HCC. A risk score using baseline clinical, laboratory, and HCC-related survival had excellent discrimination (integrated AUC: 0.71 in the validation set) and calibration (based on calibration plots and Brier scores). Models had superior performance to the BCLC and ALBI scores and similar performance to the combined BCLC-ALBI score.

**Conclusions:**

We developed a risk score using purely objective data to accurately predict long-term survival for patients with HCC. This score, if validated, can be used to prognosticate survival for patients with HCC, and, in the setting of liver transplantation, can be incorporated to consider the net survival benefit of liver transplantation versus other curative options.

## Graphical abstract

**Figure:**
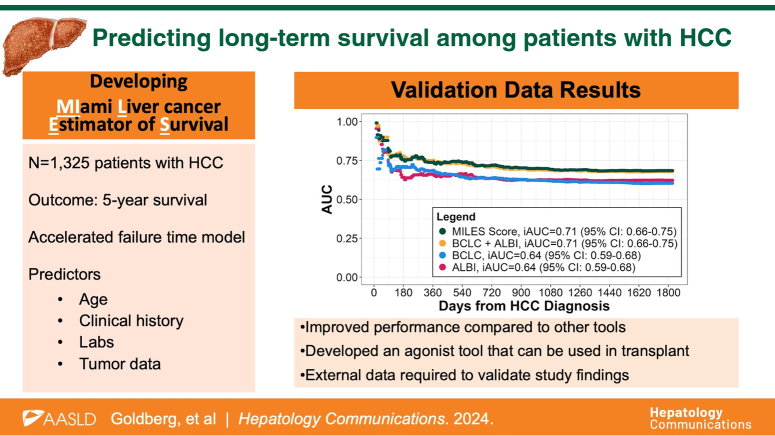

## INTRODUCTION

HCC is the sixth most common cancer worldwide and the third leading cause of cancer mortality.^1^ HCC almost always occurs in the setting of chronic liver disease (eg, hepatitis B, hepatitis C, alcohol- and metabolic syndrome–associated steatotic liver disease), and 80%–90% of patients with HCC have cirrhosis.^2^^–^^4^ As a result, HCC is unique when compared to many other cancers because the primary risk factor for HCC (cirrhosis) is also the most common cause of death (cirrhosis and liver decompensation) among patients with HCC.^5^^–^^12^ Therefore, prognosticating survival among patients with HCC and cirrhosis must account for both the tumor burden/stage as well as the severity of the underlying liver disease.^13^^–^^17^

Many staging systems for patients with HCC have been developed to prognosticate outcomes and guide treatment decision-making. The most notable include the Barcelona Clinic Liver Cancer (BCLC) classification,^13^^,^^14^ Hong Kong Liver Cancer (HKLC),^15^ and Italian Liver Cancer (ITA.LI.CA) staging systems,^17^ each of which include variables related to tumor stage and liver function; in contrast, the albumin-bilirubin (ALBI) grade only is based on liver function among patients with HCC.^18^^,^^19^ While the ITA.LI.CA has been shown to have the highest discrimination accuracy with respect to overall survival (based on the concordance index [C-index]^17^), the BCLC remains the most commonly used in clinical practice.^13^^–^^17^ Even though the BCLC (and other staging systems) are used to guide therapy, they have not been widely adopted to predict patient-level survival after the diagnosis of HCC.

For patients with HCC confined to the liver, transplantation can be curative; indeed, 10%–15% of all liver transplants are performed for HCC in the United States.^20^ With current selection criteria, tumor recurrence is infrequent (~11%), but recurrence risks are increased in those with a greater pretransplant tumor burden and/or markedly elevated tumor markers (ie, alpha-fetoprotein [AFP]).^21^^–^^24^ The liver transplantation process introduces the risk of morbidity and mortality related to the initial surgery itself, posttransplant graft complications, infections, cardiac complications, and renal dysfunction. Thus, while liver transplantation is associated with the longest survival for patients with early- or intermediate-stage HCC,^2^^–^^4^^,^^13^^,^^14^ the incremental survival benefit compared to other potentially curative therapies (eg, resection and ablation) may be as little as 1 year over a 5-year time horizon^25^^,^^26^; some patients might even be expected to suffer relative harm by pursuing transplant rather than more conservative approaches, especially when considering quality of life.^27^ Due to the persistent shortage of donor organs, individualizing the assessment of survival benefits is critical for advising patients in the clinic whether or not to pursue transplant evaluation. However, there are limited available data to estimate the survival benefit of transplantation for HCC among patients without cirrhosis who might have curative locoregional treatment options. To address this gap in knowledge, we sought to develop a risk model MIami Liver Cancer Estimator of Survival (MILES) to predict overall survival among patients with cirrhosis and early- or intermediate-stage HCC. The goal of this model would then be to optimize risk prediction among patients with cirrhosis and HCC and incorporate these predictions with posttransplant HCC prediction models to estimate expected transplant survival benefit (survival with vs. without a transplant) among patients with HCC.

## METHODS

### Study population

We conducted a retrospective cohort study among patients with cirrhosis and HCC in the Veterans Health Administration (VHA) who were included in the Veterans Outcomes and Costs Associated with Liver Disease (VOCAL) study group.^28^^–^^36^ The VHA is the largest provider of liver care in the United States, includes a comprehensive electronic medical record that includes inpatient and outpatient clinical, laboratory, imaging, and prescription data tracked across all VHA facilities, and has a diverse population that reflects the race/ethnicity distribution of the United States. The comprehensive electronic medical record provided an opportunity to abstract detailed data from radiology reports of patients receiving care at VHA facilities across the United States.^37^ In addition, because only a small fraction of patients with cirrhosis in the VHA are waitlisted for an liver transplantation (LT [either at a VHA or non-VHA transplant center]), VHA data allowed us to model the natural history of cirrhosis and HCC among a diverse cohort with less contamination (ie, censoring) of outcome data by patients undergoing a transplant.^26^^,^^38^^–^^40^

To model the survival of patients with HCC who could be eligible for a transplant: (1) we restricted our cohort to patients with cirrhosis because >95% of recipients of transplant with HCC have cirrhosis^26^; and (2) we limited analyses to patients with HCC who plausibly could be considered a candidate for transplant (eg, HCC limited to the liver without macrovascular invasion, no absolute medical contraindications to transplant [eg, heart failure with reduced ejection fraction], and Eastern Cooperative Oncology Group [ECOG] functional status ≤2).^34^^,^^41^^–^^45^ Although this led to the inclusion of patients with multifocal and/or large HCCs that would not be eligible for HCC exception points, such patients are still eligible for a transplant and may have similar outcomes to those within strict Milan criteria.^44^ Furthermore, we restricted analyses to adults aged ≥18 years and ≤75 years at cirrhosis diagnosis to generalize to the LT waitlist population because <0.3% of waitlist additions are >75 years old.^38^ Patients with an incident diagnosis of HCC from January 24, 2014, to June 9, 2021, were included with follow-up until death, liver transplantation, or end of follow-up on December 31, 2023.

Cirrhosis was defined using validated methods of 1 inpatient and/or 2 outpatient International Classification of Diseases, 9th Edition (ICD-9; 571.2 or 571.5) or ICD-10 codes (K74.60, K74. 69, K70.30, and K70.31).^28^^–^^33^ A 2-part validated method was used to identify patients with HCC: (a) query of the VHA Clinical Data Warehouse (CDW) for patients with a primary or secondary diagnosis of “malignant neoplasm of the liver” (ICD-9: 155.0; ICD-10: C22.0); and (b) manual chart abstraction of all cases by trained data abstractors.^33^ HCC was based on Liver Imaging and Reporting Data System (LiRADS) radiologic criteria and included radiology reports and/or multidisciplinary tumor board notes.^46^^–^^58^

### Data collection

Demographic, clinical, laboratory, imaging, and administrative coding data from all VHA sites were obtained from the VHA relational database, cancer data from the VHA Tumor Registry, and mortality data from the Vital Status File after approval from the Institutional Review Boards at the University of Miami, Miami VA Health System, and Corporal Michael J. Crescenz VA Medical Center. HCC data abstraction relied on radiology reports in the VHAs VistA system, HCC tumor board notes, and external imaging reports scanned into VistA, with the diagnosis of HCC requiring either a liver biopsy or a report of a Li-RADS-5 lesion from a radiology report and/or HCC Tumor Board.^59^

### Outcome

The outcome was overall survival after a confirmed diagnosis of HCC, with a focus on 1-, 3-, and 5-year survival. The index date was based on the date the patient was confirmed to have HCC based on data abstraction (ie, the first date of HCC confirmed by biopsy or a confirmed LI-RADS-5 lesion). Patient mortality was ascertained using the VA Informatics and Computing Infrastructure (VINCI) Vital Status Master File, which contained dates of death as recorded in all the main federal mortality databases. Patients were censored at the time of liver transplant.

### Predictors

The following variables, based on those at the index date, were selected a priori and evaluated for model building: age, complications of portal hypertension (ie, ascites, HE, spontaneous bacterial peritonitis, esophageal varices [varices yes/no codes due to lack of validation of codes for bleeding esophageal varices], and liver disease etiology), clinical labs (ie, AFP, international normalized ratio, sodium, albumin, platelets, total bilirubin, direct bilirubin, estimated glomerular filtration rate), and tumor data (ie, largest tumor length, total tumor diameter, the total number of tumors [LiRAD-5], ECOG functional status, and BCLC staging [0, A, B]).^26^^,^^34^^,^^41^^,^^42^^,^^44^^,^^45^ Cancer treatment was also evaluated as a binary variable. The estimated glomerular filtration rate was determined using the CKD-EPI Study equation, relying on serum creatinine levels while excluding the ethnicity coefficient.^60^^,^^61^

### Statistical analysis

The main performance metrics were the integrated area under the curve (iAUC) and the Brier score. An accelerated failure time model assuming log-logistic distribution of survival times was employed. The following distributions for accelerated failure time were tested: Weibull, Exponential, Gaussian, logistic, log-normal, and log-logistic. The distribution that fit the outcome with the lowest bayesian information criterion and Akaike information criterion was selected. The variables selected for the model were determined through backward elimination, guided by the Akaike information criterion, starting with the above-listed predictors, with the goal of maximizing the iAUC. Continuous variables were modeled using logarithmic transformation and restricted cubic splines as appropriate based on graphical summaries. The data were randomly split into training and testing subsets in a 75/25 ratio. Model performance (ie, calibration and discrimination) was evaluated in the training and testing data sets, with the final results reflecting the performance in the testing data set. The model’s discriminatory ability over different time horizons was assessed using the iAUC calculated for 1-, 3-, and 5-year survival. The accuracy of probabilistic predictions (ie, calibration) was evaluated using the integrated Brier score for 1-, 3-, and 5-year survival. In addition, calibration plots were used to visually assess the alignment between predicted survival and observed survival, utilizing a locally weighted scatterplot smoothing curve, along with metrics such as calibration-in-the-large and calibration slope. The iAUC and integrated Brier score were bootstrapped for 5000 iterations to calculate the 95% CI. A subset analysis was conducted to review the model’s accuracy for those with and without HCC treatment. We also compared the discrimination and calibration of the MILES score to the BCLC (limited to BCLC-0, A, and B), ALBI, and BCLC-ALBI scores in the test cohort, as well as those with versus without treatment. To illustrate the application of the MILES score in evaluating the survival benefit of LT, a comparison of 3-year survival outcomes with and without LT was conducted for 6 hypothetical patients. The conditional mean survival (*t* > 2.0 y) using the MILES score was calculated, representing the projected survival time for the next 3 years, assuming the patients survived long enough (2 y) to potentially receive a transplant. In essence, we accounted for the expected waiting time from diagnosis of HCC to transplant, a time period during which some patients will die or become too sick to transplant. To then account for posttransplant survival from that time point (ie, would the patient surviving 2 y after HCC diagnosis be better off continuing their management or being transplanted), we used the liver transplant expected survival HCC score to predict the restricted mean survival time for the 3 years following LT, using similar values as those imputed into the MILES score, with the exception of age, which was increased by 2 years.

To ensure we would be sufficiently powered using the proposed VHA data, we conducted a sample size analysis for time-to-event modeling, where a Cox-Snell *R* ^2^ value of 0.25 was estimated from a conservative C-statistic of 0.7. For the time-to-event model to be able to accommodate 25 predictors when the shrinkage is 0.9, the event rate is 20 per 100 patient-years, and the mean follow-up is 2.5 years over the 5-year duration, a sample size of 769 patients would have been required. In addition, to achieve 80% power in detecting an AUC of 0.7 using the DeLong test, the test data set needs to include 74 patients, assuming that 70% of patients are deceased by year 5.^62^^–^^65^ Therefore, we were confident our analyses would be sufficiently powered.

All statistical analyses were performed using R version 4.3.1 software (The R Foundation for Statistical Computing). A *p* value <0.05 was considered statistically significant. Patients were censored in the event of a liver transplant. The study was approved by the Institutional Review Board at the University of Miami and the Bruce Carter Department of Veterans Affairs Medical Center.

## RESULTS

There were 1325 patients with cirrhosis with definite HCC (Li-RADS-5 or biopsy-proven), with radiology data available for abstraction. Among the 456 (34%) patients who had no events within 5 years after HCC diagnosis, 65 were censored—45 due to transplant and 20 due to being lost to follow-up. Table 1 shows that the training and test cohorts were not different with respect to any clinical or demographic characteristics. Nearly 60% of the patients were non-Hispanic White, and nearly 25% were non-Hispanic Black, with a median age of 66 years. The most common etiologies of liver disease were HCV/alcohol-associated liver disease, followed by HCV and alcohol-associated liver disease. The majority of the patients had well-preserved synthetic function (eg, bilirubin and albumin), fewer than 15% had evidence of portal hypertension, and nearly 80% had early-stage HCC (BCLC-0 or BCLC-A). Although nearly 30% of patients did not receive any form of locoregional (eg, ablation and Y-90) or surgical treatment (eg, resection) for their HCC, there were no differences in receipt of treatment in the training and test cohorts and the 1-, 3-, and 5-year survivals were not different in the 2 groups (Table 1).

**TABLE 1:** Clinical and demographic characteristics of patients with HCC

Variables^a^	Train, N = 994	Test, N = 331
Age at HCC diagnosis	66 (62, 69)	66 (62, 69)
Sex, male, n (%)	985 (99.1)	326 (98.5)
Race/ethnicity, n (%)		
Non-Hispanic White	568 (57.1)	193 (58.3)
Non-Hispanic Black	243 (24.4)	75 (22.7)
Hispanic	70 (7.0)	29 (8.8)
Asian	25 (2.5)	6 (1.8)
Other	88 (8.9)	28 (8.5)
AFP, ng/mL	8.2 (4.1, 36.6)	9.0 (4.5, 27.1)
eGFR, mL/min/1.73 m^2^	91.3 [71.8, 98.9]	92.2 [70.5, 98.8]
INR, median	1.1 (1.0, 1.2)	1.1 (1.0, 1.2)
Sodium, mmol/L	138 [136, 140]	138 [136, 140]
Albumin, g/dL	3.7 [3.2, 4.0]	3.6 [3.3, 4.1]
Platelets, 1000/µL	140.0 [95.0, 193.0]	137.0 [91.0, 196.5]
Total bilirubin, mg/dL	0.82 (0.55, 1.35)	0.82 (0.61, 1.35)
Ascites, n (%)	41 (4.1)	15 (4.5)
HE, n (%)	28 (2.8)	7 (2.1)
Spontaneous bacterial peritonitis, n (%)	3 (0.3)	3 (0.9)
Varices, n (%)	64 (6.4)	13 (3.9)
Liver disease etiology, n (%)		
ALD + HCV	365 (36.7)	115 (34.7)
HCV	334 (33.6)	110 (33.2)
ALD	135 (13.6)	45 (13.6)
NAFLD/NASH^b^	127 (12.8)	51 (15.4)
Other	33 (3.3)	10 (3.0)
Total number of HCC tumors	1.0 (1.0, 1.0)	1.0 (1.0, 1.0)
Largest tumor (cm)	2.7 (2.0, 4.0)	2.6 (2.0, 3.8)
Total tumor size, cm	3.0 (2.1, 4.9)	3.0 (2.1, 4.7)
ECOG functional status, n (%)		
0–2	977 (98.3)	326 (98.5)
Unable to determine	17 (1.7)	5 (1.5)
BCLC, n (%)		
BCLC 0	189 (19.0)	63 (19.0)
BCLC A	584 (58.8)	199 (60.1)
BCLC B	221 (22.2)	69 (20.8)
HCC treatment, n (%)	710 (71.4)	234 (70.7)
Death at year 1, n (%)	183 (18.4)	50 (15.1)
Death at year 3, n (%)	491 (49.4)	161 (48.6)
Death at year 5, n (%)	652 (65.6)	217 (65.6)

^a^ Data reported as median (IQR) or number (%).

^b^ Data collected before the new definitions of MASLD and MASH.

Abbreviations: AFP, alpha-fetoprotein; ALD, alcohol-associated liver disease; BCLC, Barcelona Clinic Liver Cancer; ECOG, Eastern Cooperative Oncology Group; eGFR, estimated glomerular filtration rate; INR, international normalized ratio; MASH, metabolic dysfunction-associated steatohepatitis; MASLD, metabolic dysfunction-associated steatotic liver disease.

### Multivariable risk model and comparison to other models

An accelerated time failure model was used because the proportional hazard assumption for Cox regression was violated, as indicated by the Schoenfeld residual test, which showed that the hazard for key covariates changed over time. The accelerated failure time model employed a log-logistic distribution, as it had the lowest bayesian information criterion and Akaike information criterion among all the distributions analyzed. Moreover, the survival data had a heavy tail, further emphasizing it as the more appropriate distribution (Supplemental Figure S1, http://links.lww.com/HC9/B76). The survival function of the final model is listed in the Supplemental Material, http://links.lww.com/HC9/B77, and included age, international normalized ratio, sodium, albumin, total bilirubin, renal function (estimated glomerular filtration rate), AFP, history of spontaneous bacterial peritonitis or varices, and total tumor size.

**FIGURE 1:**
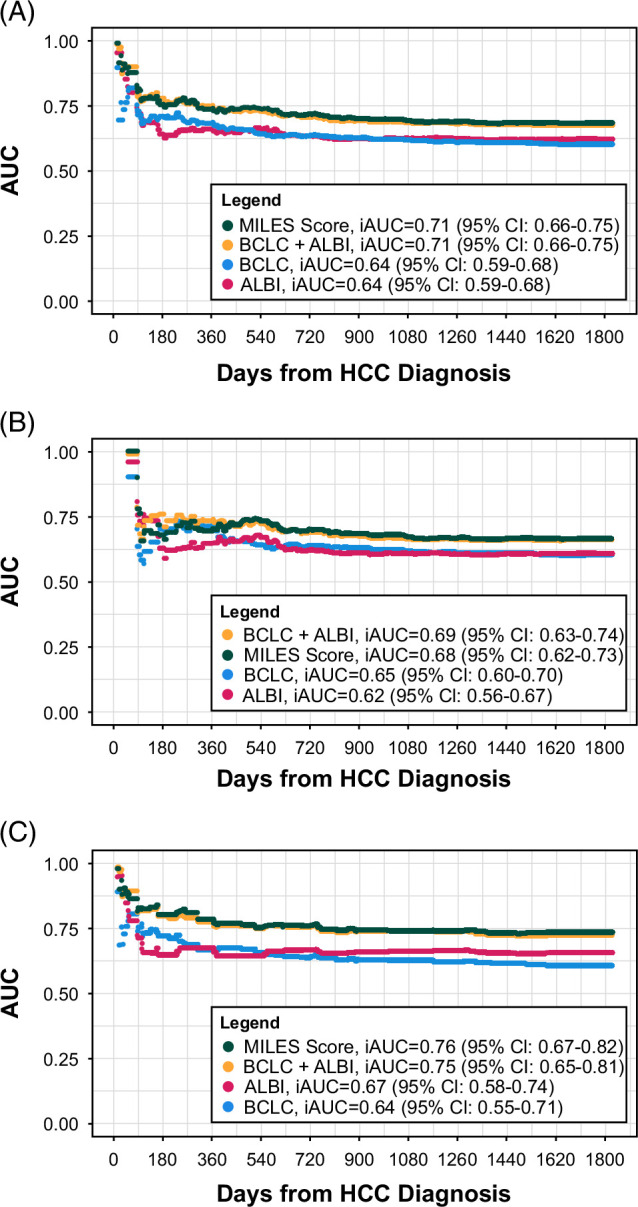
Integrated AUC of the MILES score compared to the BCLC, ALBI, and BCLC-ALBI scores. (A) Comparison of all patients in the testing data set. (B) Comparison of all patients in the testing data set who received locoregional therapy. (C) Comparison of all patients in the testing data set who did not receive locoregional therapy. Abbreviations: ALBI, albumin-bilirubin; BCLC, Barcelona Clinic Liver Cancer; iAUC, integrated area under the curve; MILES, MIami Liver Cancer Estimator of Survival.

Table 2 and Figures 1A–C show the MILES score’s discrimination. As shown, the MILEs score had superior discrimination over a 5-year time horizon compared to the BCLC score and ALBI grade, regardless of whether the entire cohort was included or only those who did (or did not) receive locoregional/surgical treatment for their HCC. However, the MILES score’s discrimination in all cohorts was not different than the BCLC-ALBI model.

**TABLE 2:** Comparison of integrated AUCs for VHA HCC risk model compared to BCLC and BCLC-ALBI models^a^

Cohort	Model	iAUC (95% CI)
All	BCLC	0.64 (0.59–0.68)
	ALBI	0.64 (0.59–0.68)
	BCLC + ALBI	0.71 (0.66–0.75)
	MILES score	0.71 (0.66–0.75)
Treated	BCLC	0.65 (0.60–0.70)
	ALBI	0.62 (0.56–0.67)
	BCLC + ALBI	0.69 (0.63–0.74)
	MILES score	0.68 (0.62–0.73)
Untreated	BCLC	0.64 (0.55–0.71)
	ALBI	0.67 (0.58–0.74)
	BCLC + ALBI	0.75 (0.65–0.81)
	MILES score	0.76 (0.67–0.82)

^a^ Discrimination of subgroups based on whether the patient received locoregional or surgical treatment of HCC during follow-up.

Abbreviations: ALBI, albumin-bilirubin; BCLC, Barcelona Clinic Liver Cancer; iAUC, integrated area under the curve; MILES, Miami Liver Cancer Estimator of Survival; VHA, Veterans Health Administration.

**FIGURE 2:**
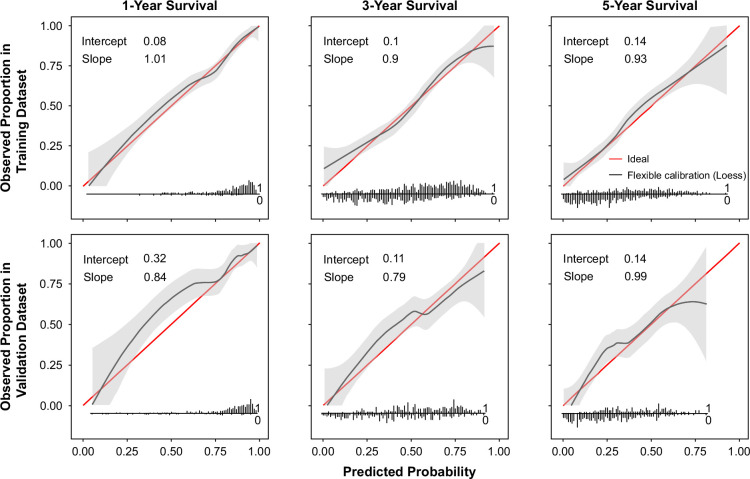
Calibration plots of the MILES score in the training and validation data sets at 1, 3, and 5 years. Abbreviation: MILES, MIami Liver Cancer Estimator of Survival.

The MILES score was well calibrated in both the training and testing data sets at the 1-, 3-, and 5-year time points (Figure 2). The Brier scores for all of the models (ie, BCLC, ALBI, BCLC-ALBI, and VHA HCC) in all cohorts (all-comers, treated, and untreated) were all good and below the ideal threshold of 0.25 (Table 3). Additional details on calibration values, including calibration-in-the-large and calibration slopes for all models, can be found in Supplemental Table S1, http://links.lww.com/HC9/B78.

**TABLE 3:** Comparison of Brier scores for VHA HCC risk model compared to BCLC and BCLC-ALBI models^a^

Cohort	Model	Brier score
All	BCLC	0.17 (0.16–0.19)
	ALBI	0.17 (0.16–0.19)
	BCLC + ALBI	0.16 (0.15–0.17)
	MILES score	0.16 (0.15–0.18)
Treated	BCLC	0.16 (0.15–0.18)
	ALBI	0.17 (0.15–0.18)
	BCLC + ALBI	0.16 (0.14–0.17)
	MILES score	0.16 (0.14–0.18)
Untreated	BCLC	0.20 (0.17–0.23)
	ALBI	0.19 (0.16–0.22)
	BCLC + ALBI	0.17 (0.14–0.20)
	MILES score	0.17 (0.14–0.20)

^a^ Discrimination of subgroups based on whether the patient received locoregional or surgical treatment of HCC during follow-up.

Abbreviations: ALBI, albumin-bilirubin; BCLC, Barcelona Clinic Liver Cancer; MILES, Miami Liver Cancer Estimator of Survival; VHA, Veterans Health Administration.

In Table 4, we highlight how the MILES score could be used to predict the survival of hypothetical patients and how this could be implemented to identify patients who might derive the greatest survival benefit from liver transplant using posttransplant survival scores such as the liver transplant expected survival HCC score.^66^ In the example shown, we provide expected 3-year survival for patients with HCC, conditioned on them surviving 2 years after diagnosis, compared to their expected 3-year posttransplant survival. In essence, this provides estimates of survival benefits for such a patient considering transplantation versus continuing nontransplant HCC management. While posttransplant survival for patients with HCC is excellent for most patients over a 3-year time horizon, even over this short time horizon, there is a range in the survival benefit of 6–12 months. In this hypothetical example, patient 6 would derive a 1-year survival benefit with transplant over a 3-year time horizon, compared with only a 6-month (0.5-y) benefit for patient 1. Despite these differences, under the US liver allocation system, all 6 patients would receive the same waitlist priority by virtue of having HCC within Milan criteria.^20^

**TABLE 4:** Comparison of predicted survival for 6 sample patients with newly diagnosed HCC

Variables in MILES score	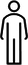	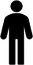	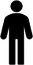	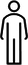	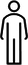	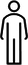
Age at HCC diagnosis	68	53	59	49	71	61
Total bilirubin	1.2	1.8	1.4	2.8	1.6	2.1
INR	1.1	1.3	1.2	1.5	1.2	1.3
Sodium	138	134	133	132	139	135
Albumin	3.8	3.6	3.4	3.3	3.9	3.7
eGFR	90	80	60	45	85	70
Diabetes	Yes	No	Yes	No	No	No
SBP	No	No	No	No	No	No
Chronic kidney disease	No	No	Yes	Yes	No	No
Esophageal varices	No	Yes	No	Yes	Yes	No
Etiology of liver disease	NASH	Alcohol	Alcohol	HBV	Other	Other
AFP	15	5	25	50	10	100
Total tumor diameter	3	4	3	2	2	4
Ventilated before transplant	No	No	No	No	No	No
Pretransplant location	Home	Home	Home	Home	Home	Home
MILES estimated mean survival conditional on 2-y survival^a^	2.1	2.0	1.8	1.8	2.0	1.7
LiTES-Score	24.1	36.5	23.2	43.4	23.9	30.9
LiTES-Score estimated mean survival^a^	2.6	2.8	2.7	2.7	2.6	2.7

^a^ Predicted survival time (years) based on 3-year horizon. Assumed values at listing and pretransplant were the same.

Abbreviations: AFP, alpha-fetoprotein; eGFR, estimated glomerular filtration rate; INR, international normalized ratio; LiTES, liver transplant expected survival; MILES, MIami Liver Cancer Estimator of Survival; SBP, spontaneous bacterial peritonitis.

## DISCUSSION

Using clinical, laboratory, and HCC-specific variables, we developed the MILES score to predict survival from the time of diagnosis in a diverse cohort with early- and intermediate-stage HCC. The model has good discrimination and calibration in all-comers, regardless of receipt of locoregional treatment for their HCC. This score had superior performance to the BCLC classification system, the most commonly employed HCC prognostic system, and similar performance to the BCLC-ALBI score. If validated in an external cohort, this score could be used to help estimate the long-term survival benefit of transplantation for patients with HCC, thereby helping to transform the paradigm by which transplant is considered in the treatment algorithm for patients with HCC.

Our scoring system is not the first to predict survival among patients with HCC. While the BCLC classification, HKLC, ITA.LI.CA staging system, and ALBI grade also predict survival among patients with HCC, they are largely used to help guide therapy rather than to provide individual-level predictions of survival.^13^^–^^15^^,^^17^^–^^19^ Importantly, all of these scores have been developed and validated in all-comers with HCC, including those with advanced disease, while the MILES score cohort was restricted to adults meeting broad criteria for liver transplantation.

BCLC classification, HKLC, and ITA.LI.CA staging systems have the key limitation of comprising 1 or more subjective measures. Both the HKLC (derived from Asian patients who primarily had HBV in the absence of cirrhosis) and ITA.LI.CA include Child-Turcotte-Pugh score in their staging, which includes subjective components that are subject to interprovider variability (ie, encephalopathy and ascites stage)^67^^,^^68^ In addition, the BCLC, HKLC, and ITA.LI.CA include ECOG functional status, a subjective measure with demonstrated suboptimal inter-rater reliability.^69^^–^^71^ In addition, the BCLC model places a great deal of emphasis on ECOG, a subjective measure, leading it to categorize patients with a small single HCC and preserved liver function (eg, Child Class A) but a functional status of ECOG 1 as BCLC-C (ie, advanced stage). For such a patient, the BCLC would recommend systemic treatment and against transplant, even though an ECOG functional status of 1 would not be a contraindication to a liver transplant.^13^^,^^72^ Furthermore, the inclusion of a subjective variable makes these models ill-suited in the context of a risk score for transplant allocation, especially in the United States, where the federal regulatory framework requires patients to be prioritized based on “objective and measurable medical criteria.”^15^^,^^17^^,^^73^^,^^74^ Objections to including a subjective measure in transplant data collection are not just theoretical but raise ethical concerns as the history of liver transplant allocation, unfortunately, contains occasional examples of abuse by centers motivated to advance their patients’ priority.^75^ The Karnofsky score data were used for center-specific risk adjustment in the United States, but this practice was discontinued as the data reported in the transplant registry were unreliable and subject to bias. In addition to the inclusion of subjective data, these other risk scores relied on binary cutpoints for continuous variables (eg, tumor size and AFP).^13^^–^^17^^,^^76^^–^^78^ While the MILES score has similar discrimination to the combined BCLC-ALBI score, it is a more attractive option to predict survival for patients with early- or intermediate-stage HCC because it is based purely on objective variables treated in a continuous fashion, which would be especially important if this model were incorporated into survival benefit–based liver allocation in the future.^76^^–^^78^ Furthermore, with the exception of varices, all the data in the MILES score are already routinely collected in the US transplant registry, which would minimize the need for substantially increased data collection if the score was used for liver allocation.

In Table 4, we present how the MILES score could be applied in clinical practice and used in treatment decision-making and organ allocation. In the setting of transplantation for patients with HCC, for which transplant is not the only curative treatment option, it is important to consider the survival benefit of transplant. On the individual patient level, these data could be used to counsel patients considering transplants and whether they would be expected to derive a survival benefit from transplantation versus other treatment options. On the public health/policy level, incorporating this score could help to prioritize patients who derive the greatest survival benefit with transplants. Under a survival benefit–based allocation system, the score could be used to prioritize patients waitlisted with HCC. Or, at the very least, survival benefit could be used as the tiebreaker for prioritization of patients with HCC exceptions and the same MELD score, replacing the current waiting time used to differentiate patients with the same exception score.

We acknowledge the study limitations. We relied on data from the VHA, which is mostly comprised of men and may not be fully representative of the US population. However, the HCC-specific survival does not differ for the most common etiologies of cirrhosis and HCC in our cohort (eg, alcohol) compared to the emerging leading cause (Metabolic dysfunction-associated steatotic liver disease/Metabolic dysfunction-associated steatohepatitis), thereby giving confidence our model will have external validity.^79^ Second, we excluded patients who had imaging outside the VHA system that did not have results available in VISTA. However, we would not have expected the exclusion of these patients to bias our results because scanning of outside images into VISTA should not be related to the severity of HCC. Third, predictions regarding LT survival benefit assumed lab values at the time of transplant to be the same as baseline, which may not be reflective of what happens with patients. Fourth, we were unable to determine the cause of death, a limitation of any retrospective cohort study without a detailed medical record review. Lastly, our model focused only on predicting survival based on data available at the time of diagnosis rather than being a time-varying model. Future work can refine the model to account for updated covariates, but our objective in this study was specifically to model survival from diagnosis, the key time point at which decisions about initial treatment are required.

In conclusion, we developed a risk score that uses objective data to predict survival for patients with cirrhosis and HCC. Our model performed as well as, if not better than, existing HCC survival models while having the benefit of including only objective variables. If externally validated, this model could be incorporated into clinical practice and, in the future, be included in a survival benefit–based liver allocation model that accounts for survival with versus without a transplant to better ration the scarce resource of donor livers.

## Supplementary Material

**Figure:**