# Supplementary material for: Predicting long-term survival among patients with HCC
Source: Hepatol Commun. 2024 Nov 4;8(11):e0581. doi: 10.1097/HC9.0000000000000581 (PMC11537595; doi:10.1097/HC9.0000000000000581)
Supplement: Supplementary file 1 [file hc9-8-e0581-s002.docx]

Supplementary material: Survival function of the final model

$S\left( t \right)=\frac{1}{1+(\lambda{t)}^{\frac{1}{0.5753}}}$ where

$\lambda\theta\theta=exp(-[\beta_{age}- 0.1241\ln AFP+\beta_{eGFR}- 0.4650\ln INR+0.0325 Sodium+0.5298 Albumin-0.1767 Total Bilirubin- 0.9848 SBP- 0.2256 Varices-0.4419\ln Total Tumor Size])$,

$\beta_{age}=\left\{ \begin{aligned} \begin{matrix} 6.1056-0.0902 age, & age<40 \end{matrix} \\ \begin{matrix} 2.689+0.1661age-0.006405 {age}^{2}+{0.00005338 age}^{3}, & 40>age>64 \end{matrix} \\ \begin{matrix} 350.7353+16.1486 age+0.2485 {age}^{2}-{0.001274 age}^{3}, & 64>age>68 \end{matrix} \\ \begin{matrix} -221.3674+9.0912 age-0.12266 {age}^{2}+{0.00054517 age}^{3}, & 68>age>75 \end{matrix} \\ \begin{matrix} 8.6255-0.1085 age, & age>75 \end{matrix} \end{aligned} \right.$,

$$\beta_{eGFR}=\left\{ \begin{aligned} \begin{matrix} 6.7843+0.007204 eGFR, & eGFR<4.2538 \end{matrix} \\ \begin{matrix} 6.7844+0.007194 eGFR+2.261{x10}^{-6} {eGFR}^{2}-{1.772{x10}^{-7} eGFR}^{3}, & 4.2538>eGFR>78.0884 \end{matrix} \\ \begin{matrix} 13.8719-0.2651 eGFR+0.003489 {eGF R}^{2}-{1.506{x10}^{-5} eGFR}^{3}, & 78.0884>eGFR>96.3980 \end{matrix} \\ \begin{matrix} -6.8406+0.3795 eGFR-0.003198 {eGFR}^{2}+{{8.061x10}^{-6} eGFR}^{3}, & 96.3980>eGFR>132.2331 \end{matrix} \\ \begin{matrix} 11.7968-0.04333 eGFR, & eGFR>132.2331 \end{matrix} \end{aligned} \right.$$
