# Supplementary material for: Predicting long-term survival among patients with HCC
Source: Hepatol Commun. 2024 Nov 4;8(11):e0581. doi: 10.1097/HC9.0000000000000581 (PMC11537595; doi:10.1097/HC9.0000000000000581)
Supplement: Supplementary file 2 [file hc9-8-e0581-s003.docx]

Supplement Table 1. Calibration-in-the-large values and calibration slope for all the models including values when subset by those with and without HCC treatment

| **Cohort** | **Model** | **Year 1** | | **Year 3** | | **Year 5** | |
| --- | --- | --- | --- | --- | --- | --- | --- |
|  |  | **CITL** | **Slope** | **CITL** | **Slope** | **CITL** | **Slope** |
| All | MILES | 0.32 | 0.84 | 0.11 | 0.79 | 0.14 | 0.99 |
|  | BCLC | 0.28 | 1.45 | 0.10 | 1.10 | 0.11 | 1.19 |
|  | ALBI | 0.31 | 0.93 | 0.10 | 0.94 | 0.10 | 1.13 |
|  | BCLC + ALBI | 0.33 | 1.16 | 0.10 | 1.01 | 0.10 | 1.18 |
| Treated | MILES | 0.72 | 0.73 | 0.18 | 0.67 | 0.10 | 0.88 |
|  | BCLC | 0.68 | 1.49 | 0.18 | 0.96 | 0.09 | 1.20 |
|  | ALBI | 0.70 | 0.76 | 0.16 | 0.76 | 0.06 | 0.96 |
|  | BCLC + ALBI | 0.73 | 1.05 | 0.16 | 0.84 | 0.05 | 1.07 |
| Untreated | MILES | -0.39 | 1.02 | -0.06 | 1.15 | 0.23 | 1.32 |
|  | BCLC | -0.40 | 1.50 | -0.08 | 1.47 | 0.16 | 1.18 |
|  | ALBI | -0.35 | 1.18 | -0.05 | 1.45 | 0.20 | 1.61 |
|  | BCLC + ALBI | -0.33 | 1.37 | -0.04 | 1.52 | 0.22 | 1.47 |

Abbreviations: CITL=calibration-in-the-large, Slope=calibration slope
